# Karyotype diversity of pseudoscorpions of the genus *Chthonius* (Pseudoscorpiones, Chthoniidae) in the Alps

**DOI:** 10.3897/CompCytogen.v10i3.8906

**Published:** 2016-08-31

**Authors:** Jana Kotrbová, Vera Opatova, Giulio Gardini, František Šťáhlavský

**Affiliations:** 1 Charles University in Prague, Faculty of Science, Department of Zoology, Viničná 7, CZ-12844 Praha, Czech Republic; 2 c/o DISTAV, Università degli Studi, Genova, corso Europa 26, I-16132 Genova, Italy

**Keywords:** Karyotype evolution, chromosome fusion, sex chromosomes, X0 sex chromosome system, achiasmatic meiosis

## Abstract

Pseudoscorpions are found in almost all terrestrial habitats. However, their uniform appearance presents a challenge for morphology-based taxonomy, which may underestimate the diversity of this order. We performed cytogenetic analyses on 11 pseudoscorpion species of the genus *Chthonius* C. L. Koch, 1843 from the Alps, including three subgenera: Chthonius (Chthonius) C. L. Koch, 1843, Chthonius (Ephippiochthonius) Beier, 1930 and Chthonius (Globochthonius) Beier, 1931 inhabiting this region. The results show that the male diploid number of chromosomes ranges from 21–35. The sex chromosome system X0 has been detected in all male specimens. The X sex chromosome is always metacentric and represents the largest chromosome in the nucleus. Achiasmatic meiosis, already known from the family Chthoniidae, was further confirmed in males of *Chthonius*. C-banding corroborated the localization of constitutive heterochromatin in the centromere region, which corresponds to heteropycnotic knobs on the standard chromosome preparations. Morphological types and size differentiation of chromosomes in the karyotype suggest that the main chromosomal rearrangements in the evolution of *Chthonius* are centric or tandem fusions resulting in a decrease in the number of chromosomes. Pericentric inversions, inducing the change of acrocentric chromosomes into biarmed chromosomes, could also be expected. Variability in chromosome morphology and number was detected in several species: Chthonius (Chthonius) ischnocheles (Hermann, 1804), Chthonius (Chthonius) raridentatus, Chthonius (Chthonius) rhodochelatus Hadži, 1930, and Chthonius (Chthonius) tenuis L. Koch, 1873. We discuss the intraspecific variability within these species and the potential existence of cryptic species.

## Introduction

Pseudoscorpions are the fourth most numerous order of the class Arachnida, comprising 3385 described species currently classified into 439 genera and 26 families (Harvey 2013). However, the true diversity of the order might be underestimated due to the challenging morphology of the group in addition to the very small body size of individuals (usually not exceeding 2 mm). The taxonomy of pseudoscorpions is often based on character states with poorly defined variability. For instance, the range of frequently used measurements and counts of specific setae may differ significantly depending on the number of specimens analysed per species. Furthermore, overlaps in species-specific character states complicate the exact identification of many species complexes (compare e.g. Beier 1963, Christophoryová et al. 2011, Gardini 2013, 2014). Molecular techniques have further revealed the limitations of traditional morphology-based classification of pseudoscorpions. Despite the absence of molecular techniques in formal species delimitation in this group, independent lineages that may correspond to cryptic species have been detected in number of cases (e.g. Wilcox et al. 1997, Moulds et al. 2007, Pfeiler et al. 2009, van Heerden et al. 2013, Harrison et al. 2014). Another useful method for detecting unaccounted diversity is karyotype analysis. Significant interspecific differences in karyotypes may reveal distinct lineages constituting cryptic species in some morphologically challenging groups (e.g. Řezáč et al. 2007, Lukhtanov et al. 2015). In pseudoscorpions, the use of cytogenetic methods has enabled the detection of interspecific variability (Troiano 1990, Šťáhlavský et al. 2006, 2013) and has led to the description of a new species (Zaragoza and Šťáhlavský 2008).

Currently, there is karyotype information for about 51 species belonging to 25 genera from eight families (Atemnidae, Garypinidae, Geogarypidae, Cheliferidae, Chernetidae, Chthoniidae, Neobisiidae, Olpiidae) (Šťáhlavský 2016). The chromosome number among pseudoscorpions ranges from 2n = 7–143 (Šťáhlavský 2016), but the variability of the chromosome number tends to be specific for each group. Despite the previous success of using karyological differences for species delimitation in pseudoscorpions (Zaragoza and Šťáhlavský 2008), this approach is limited due to the lack of cytogenetic data for comparison. Most of the available data belongs to both geographically and evolutionary distant lineages, where major differences in karyotype are not surprising. Often, only a few specimens were sampled per species/population, resulting in a lack of information about intraspecific variability (e.g. Šťáhlavský et al. 2006, 2009, 2012). In order to shed light on the karyotype differentiation among more closely related species and to enhance our knowledge on intraspecific karyotype variability, we focus on cytogenetic analyses of the genus *Chthonius* from the alpine region.

The genus *Chthonius* comprises 260 described species, mainly inhabiting leaf litter (Harvey 2013). In the Alps, *Chthonius* is represented by 44 species classified into three subgenera: *Ephippiochthonius*, *Chthonius* and *Globochthonius* (Gardini 2013, 2014). Several species have been newly described, or taken from the synonymy during the recent morphological revision of the genus (Gardini 2013, 2014). New faunistic data also suggest that the distribution of species ranges in the Alps may be different from previously thought (Gardini 2000).

Due to altitudinal zonation, high mountain regions such as the Alps offer a wide range of habitats and generally present high species richness. High levels of diversity and endemism are traditionally explained by the geographic isolation of organisms with specific ecological preferences in relatively small areas and distribution range shifts during periods of glaciation (Schmitt 2009). These two factors may have significant effects on organisms with low dispersal potential, where geographic isolation may lead to karyotype differentiation that subsequently presents an effective reproductive barrier (e.g. King 1993, Kawakami et al. 2011). Sedentary organisms such as ground dwelling pseudoscorpions of the genus *Chthonius* are excellent models for cytogenetic studies within the context of this ecologically diverse region with a dynamic climatic history.

## Material and methods

Individuals used in the present study were obtained from leaf litter sifting or were collected individually under stones. The collection data for the species used in this study are listed below. After the name of each species, the information is lined-up in brackets as following: total number of analysed specimens / total number of analysed cells / total number of measured cells.

Chthonius (Chthonius) alpicola Beier, 1951 (2/16/5): Italy: Forni di Sotto (46.399 N, 12.689 E), 1 ♀; Italy: Santa Caterina (46.512 N, 13.395 E), 1 ♂.

Chthonius (Chthonius) carinthiacus Beier, 1951 (7/31/5): Italy: Lago di Ledro (45.866 N, 10.741 E), 1 ♂; Italy: Passo Cereda (46.194 N, 11.914 E), 1 ♂; Italy: Tarvisio (46.527 N, 13.545 E), 2 ♂; Italy: Tramonti di Sopra (46.353 N, 12.783 E), 1 ♂; Italy: Vittorio Veneto (45.983 N, 12.283 E), 1 ♂; Slovenia: Bohinjska Bistrica (46.279 N, 13.962 E), 1 ♂.

Chthonius (Chthonius) ischnocheles (Hermann, 1804), cytotype I (2/27/5): France: Glère (47.342 N, 06.971 E), 1 ♂; Switzerland: Bieane (47.123 N, 07.208 E), 1 ♂.

Chthonius (Chthonius) ischnocheles (Hermann, 1804), cytotype II (13/120/10): Switzerland: Valangin (47.016 N, 06.908 E), 1 ♂; Italy: Castello (46.027 N, 09.046 E), 1 ♂; Italy: Egna (46.313 N, 11.290 E), 2 ♂; Italy: Lebenberg (46.640 N, 11.135 E), 1 ♂; Italy: Lichtenberg (46.632 N, 10.564 E), 3 ♂; Italy: Pannone (45.871 N, 10.933 E), 1 ♂; Italy: Vermiglio (46.290 N, 10.678 E), 4 ♂.

Chthonius (Chthonius) raridentatus Hadži, 1930, cytotype I (35/169/129): Italy: Tramonti di Sopra (46.353 N, 12.783 E), 1 ♀; Slovenia: Kamnik (46.224 N, 14.614 E), 1 ♂; Slovenia: Kamniška Bistrica (46.310 N, 14.601 E), 1 ♂; Slovenia: over Bohinska Bistrica (46.276 N, 14.007 E), 1 ♂; Slovenia: Roče (46.108 N, 13.816 E), 31 ♂.

Chthonius (Chthonius) raridentatus Hadži, 1930, cytotype II (4/105/15): Austria: Barenhtal (46.482 N, 14.170 E), 1 ♂; Slovenia: Bohinjska Bistrica (46.279 N, 13.962 E), 2 ♂; Slovenia: Roče (46.108 N, 13.816 E), 1 ♂.

Chthonius (Chthonius) rhodochelatus Hadži, 1933, cytotype I (5/81/8): Italy: Lago di S. G. Sanzena (46.357 N, 11.069 E), 1 ♂; Italy: Loppio (45.859 N, 10.924 E), 1 ♂; Italy: Nuova Olomio (46.161 N, 09.433 E), 1 ♂; Italy: Puria (46.033 N, 09.049 E), 1 ♂; Italy: Sondrio (46.175 N, 09.857 E), 1 ♂.

Chthonius (Chthonius) rhodochelatus Hadži, 1933, cytotype II (1/32/6): 1 ♂; Italy: Lago di S. G. Sanzena (46.357 N, 11.069 E), 1 ♂.

Chthonius (Chthonius) tenuis L. Koch, 1873, cytotype I (37/514/10): Italy: Buisson (45.837 N, 07.605 E), 2 ♂; Italy: Cannobio (46.059 N, 08.699 E), 1 ♂; Italy: Carona (46.017 N, 09.780 E), 3 ♂; Italy: Dezzo di Scalve (45.974 N, 10.104 E), 5 ♂; Italy: Forte di Bard (45.606 N, 07.744 E), 1 ♂; Italy: Imperia (43.939 N, 07.829 E), 2 ♂; Italy: Isoladi Fondra (45.966 N, 09.734 E), 1 ♂; Italy: Loreglia (45.902 N, 08.370 E), 2 ♂; Italy: Melle (44.560 N, 07.314 E), 1 ♂; Italy: Noli (44.200 N, 08.405 E), 2 ♂, 1♀; Italy: Pont-Saint-Martin (45.607 N, 07.810 E), 1 ♂; Italy: Puria (46.033 N, 09.049 E), 1 ♂; Italy: Sondrio (46.175 N, 09.857 E), 2 ♂; Italy: Trarego Viggiona (46.042 N, 08.652 E), 3 ♂; Italy: Vermiglio (46.290 N, 10.678 E), 1 ♂; Italy: Zambla (45.877 N, 09.777 E), 2 ♂; Switzerland: Engelberg (46.828 N, 08.413 E), 4 ♂; Switzerland: Mauracker (46.279 N, 07.813 E), 2 ♂.

Chthonius (Chthonius) tenuis L. Koch, 1873, cytotype II (1/34/8): Slovenia: over Bohinska Bistrica (46.276 N, 14.007 E), 1 ♂.

Chthonius (Chthonius) tenuis L. Koch, 1873, cytotype III (1/19/8): Austria: Altfinkenstein (46.548 N, 13.876 E), 1 ♂.

Chthonius (Chthonius) tenuis L. Koch, 1873, cytotype IV (1/8/8): Italy: Pont-Saint-Martin (45.607 N, 07.810 E), 1 ♂.

Chthonius (Chthonius) tenuis L. Koch, 1873, cytotype V (2/17/8): Italy: Noli (44.200 N, 08.405 E), 2 ♂.

Chthonius (Ephippiochthonius) boldorii Beier, 1934 (11/120/10): Austria: Altfinkenstein (46.548 N, 13.876 E), 2 ♂; Austria: Saak (46.592 N, 13.626 E), 2 ♂; Austria: Tscheppachslucht (46.503 N, 14.284 E), 2 ♂; Switzerland: Somazzo (45.884 N, 08.992 E), 1 ♂; Italy: Loppio (45.859 N, 10.924 E), 1 ♂; Italy: Mezzoldo (46.015 N, 09.665 E), 1 ♂; Italy: Puria (46.033 N, 09.049 E), 1 ♂; Italy: Vittorio Veneto (45.983 N, 12.283 E), 1 ♂.

Chthonius (Ephippiochthonius) fuscimanus Simon, 1900 (2/26/8): Italy: Selva di Cerda (46.445 N, 12.024 E), 2 ♂.

Chthonius (Ephippiochthonius) nanus Beier, 1953 (3/21/6): Italy: Imperia (43.939 N, 07.829 E), 3 ♂.

Chthonius (Ephippiochthonius) tetrachelatus (Preyssler, 1790) (3/67/14): Austria: Altfinkenstein (46.548 N, 13.876 E), 1 ♂; Austria: Vittorio Veneto (45.983 N, 12.283 E), 1 ♂; Slovenia: Srpenica (46.295 N, 13.493 E), 1 ♂.

Chthonius (Globochthonius) poeninus Mahnert, 1979 (1/44/6): Italy: Castello (46.027 N, 09.046 E), 1 ♂.

Chromosome preparations were obtained by the “plate spreading” method (Traut 1976), which has been successfully applied on the genus *Chthonius* (Šťáhlavský and Král 2004). Male gonads used in this protocol were immersed into hypotonic solution of 0.075 M KCL for 20 min and subsequently fixated in methanol: acetic acid (3:1) solution for 20 min. Fixed tissue was transferred onto a microscope slide, the cell dissociated and spread in a drop of 60% acetic acid on the histological plate (40-45 ºC). Chromosome preparations were stained in a 5% Giemsa solution in Sörensen phosphate buffer for 30 min (Šťáhlavský and Král 2004). Constitutive heterochromatin was visualised by C-banding, following the standard protocol (Sumner 1972) on selected preparations of seven males of *Chthonius raridentatus* and one male of *Chthonius tetrachelatus*. Chromosome preparations were observed in Olympus AX70 Provis microscope and documented with an Olympus DP71 camera. Frequently, the centromeres were indistinct during the mitotic metaphases, thus cells at pachytene, postpachytene, metaphase I or metaphase II with a clearly distinct centromere position were used for karyotype analyses. Photographed chromosomes were checked for standard karyotype characteristics such as number, relative size, and morphology of the chromosomes using LEVAN plugin (Sacamato and Zacaro 2009) for IMAGEJ 1.47 program (http://imagej.nih.gov/ij/), which allows a direct classification of chromosomal types and their relative size calculation. Morphology of the chromosomes was determined following standard classification (Levan et al. 1964). Relative length of chromosomes (RCL) was calculated for a haploid set including the X sex chromosome. In *Chthonius raridentatus* cytotype I from Roče (Slovenia), given the abundance of dividing cells, we applied t-test using software STATISTICA 9.0 (www.statsoft.com) to determine whether the measurements of chromosomes are significantly different (the threshold chosen for statistical significance α = 0.05) during distinct spiralization of chromosomes of various meiotic (postpachytene, metaphase I, metaphase II) and mitotic (mitotic metaphase) stages (Suppl. material 1).

## Results

Karyology data were obtained for 11 species of pseudoscorpions from the genus *Chthonius* (Chthoniidae) (Table 1) comprising Chthonius (Chthonius), Chthonius (Ephippiochthonius) and Chthonius (Globochthonius) subgenera.

**Table 1.** Summary of the cytogenetic data for the genus *Chthonius*.

		Sex	Morphology of autosomes					
**Species**	**2n**	**chrom.**	**M**	**SM**	**ST**	**A**	**Country**	**References**
Chthonius (Chthonius) alpicola	21	X0				20	IT	present study
Chthonius (Chthonius) carinthiacus	35	X0				34	CZ, IT	Šťáhlavský and Král 2004, present study
Chthonius (Chthonius) heterodactylus	33	X0		4		28	RO	Šťáhlavský and Král 2004
Chthonius (Chthonius) ischnocheles, cytotype I	31	X0	4			26	CH, FR	present study
Chthonius (Chthonius) ischnocheles, cytotype II	35	X0	4	2		28	CH, IT	present study
Chthonius (Chthonius) litoralis	35	X0			2	32	GR	Šťáhlavský and Král 2004
Chthonius (Chthonius) orthodactylus	33	X0		2		30	CZ	Šťáhlavský and Král 2004
Chthonius (Chthonius) raridentatus, cytotype I	29	X0	2		2	24	SI	present study
Chthonius (Chthonius) raridentatus, cytotype II	29	X0	4			24	SI	present study
Chthonius (Chthonius) rhodochelatus, cytotype I	35	X0	4			30	IT	present study
Chthonius (Chthonius) rhodochelatus, cytotype II	35	X0	2	2		30	IT	present study
Chthonius (Chthonius) tenuis, cytotype I	35	X0	2			32	CH, IT	present study
Chthonius (Chthonius) tenuis, cytotype II	33	X0	2			30	SI	present study
Chthonius (Chthonius) tenuis, cytotype III	33	X0	4		2	26	AT	present study
Chthonius (Chthonius) tenuis, cytotype IV	33	X0				32	IT	present study
Chthonius (Chthonius) tenuis, cytotype V	21	X0	6			14	IT	present study
Chthonius (Ephippiochthonius) boldorii	35	X0			2	32	AT, IT	present study
Chthonius (Ephippiochthonius) fuscimanus	35	X0				34	CZ, IT	Šťáhlavský and Král 2004, present study
Chthonius (Ephippiochthonius) tetrachelatus, cytotype I	35	X0		2		32	CZ	Šťáhlavský and Král 2004
Chthonius (Ephippiochthonius) tetrachelatus, cytotype II	35	X0		2	2	30	SI	present study
Chthonius (Ephippiochthonius) nanus	25	X0	2			22	IT	present study
*C. (E.) sp. 1*	29	X0	2	4		22	GR	Šťáhlavský and Král 2004
*C. (E.) sp. 2*	21	X0	4	2	2	12	GR	Šťáhlavský and Král 2004
Chthonius (Globochthonius) poeninus	25	X0	2			22	IT	present study

Abbreviations: A – acrocentric , AT – Austria , CH – Switzerland , CZ – Czech Republic , FR – France , GR – Greece , IT – Italy , M – metacentric , RO – Romania , SI – Slovenia , SM – submetacentric , ST – subtelocentric

### Chthonius (Chthonius) alpicola Beier, 1951

**Figure 1.**
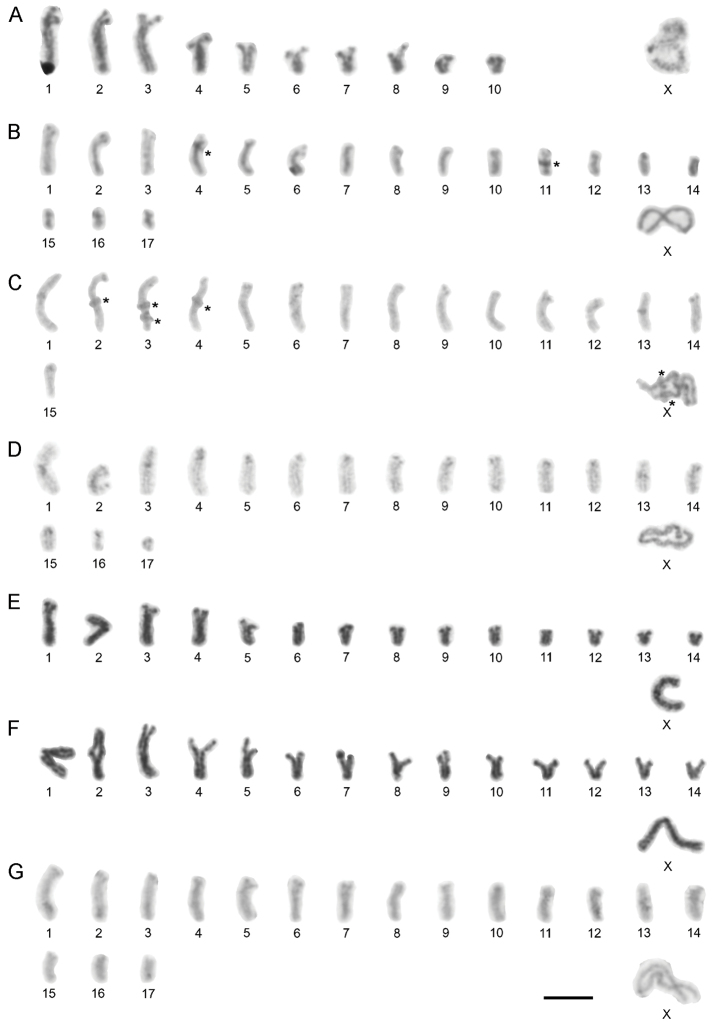
Karyotypes of Chthonius (Chthonius) males based on postpachytene and metaphase I. **A** Chthonius (Chthonius) alpicola (2n = 21, X0) (large black spot on the first autosome pair represents overlap with the sperm) **B** Chthonius (Chthonius) carinthiacus (2n = 35, X0) **C** Chthonius (Chthonius) ischnocheles, cytotype I (2n = 31, X0) **D** Chthonius (Chthonius) ischnocheles, cytotype II (2n = 35, X0) **E** Chthonius (Chthonius) raridentatus, cytotype I (2n = 29, X0) **F** Chthonius (Chthonius) raridentatus, cytotype II (2n = 29, X0) **G** Chthonius (Chthonius) rhodochelatus, cytotype II (2n = 35, X0). Asterisks indicate chromosome overlaps. Scale bar = 10 µm.

The diploid set consists of 21 chromosomes in male (Fig. 1a) and 22 chromosomes in female. The male karyotype comprises ten pairs of acrocentric autosomes and one metacentric X sex chromosome. The first three acrocentric pairs of autosomes are significantly longer (RCLs 13.23%, 12.49% and 10.92%) than the remaining autosome pairs that gradually decrease in RCL from 7.98% to 2.97%. The X represents the largest chromosome in the karyotype reaching the length of 27.36% of the haploid set.

### Chthonius (Chthonius) carinthiacus Beier, 1951

Seven individuals from central and eastern parts of the Alps displayed 2n = 35 in all cases (Fig. 1b). The karyotype of this species comprises 17 pairs of acrocentric autosomes and one metacentric X sex chromosome. The RCL of autosomes gradually decreases from 7.29% to 2.34%. The RCL of the X chromosome is 23.30%.

### Chthonius (Chthonius) ischnocheles (Hermann, 1804)

Variability in chromosome number and morphology was detected in this species; two different cytotypes were distinguished. Cytotype I was detected only in two males from two geographically proximate localities in Switzerland and France. The diploid set of this cytotype comprises 31 chromosomes (Fig. 1c). There are 13 pairs of acrocentric and two pairs of metacentric autosomes (pairs No. 1 and 13) and one metacentric X chromosome. Autosome RCLs decrease gradually from 8.47% to 3.66%. The RCL of the X is 17.85%. Cytotype II was detected from seven different localities. The diploid number of chromosomes of this cytotype is 35 (Fig. 1d). The karyotype comprises 14 pairs of acrocentric autosome pairs, two metacentric pairs (pairs No. 1 and 13), and one submetacentric pair (pair No. 3), with the X chromosome metacentric. The RCLs of the autosomes decrease gradually from 7.47% to 1.45% and the last autosome is significantly shorter than the previous pair. The RCL of the X is 17.12%.

### Chthonius (Chthonius) raridentatus Hadži, 1930

**Figure 2.**
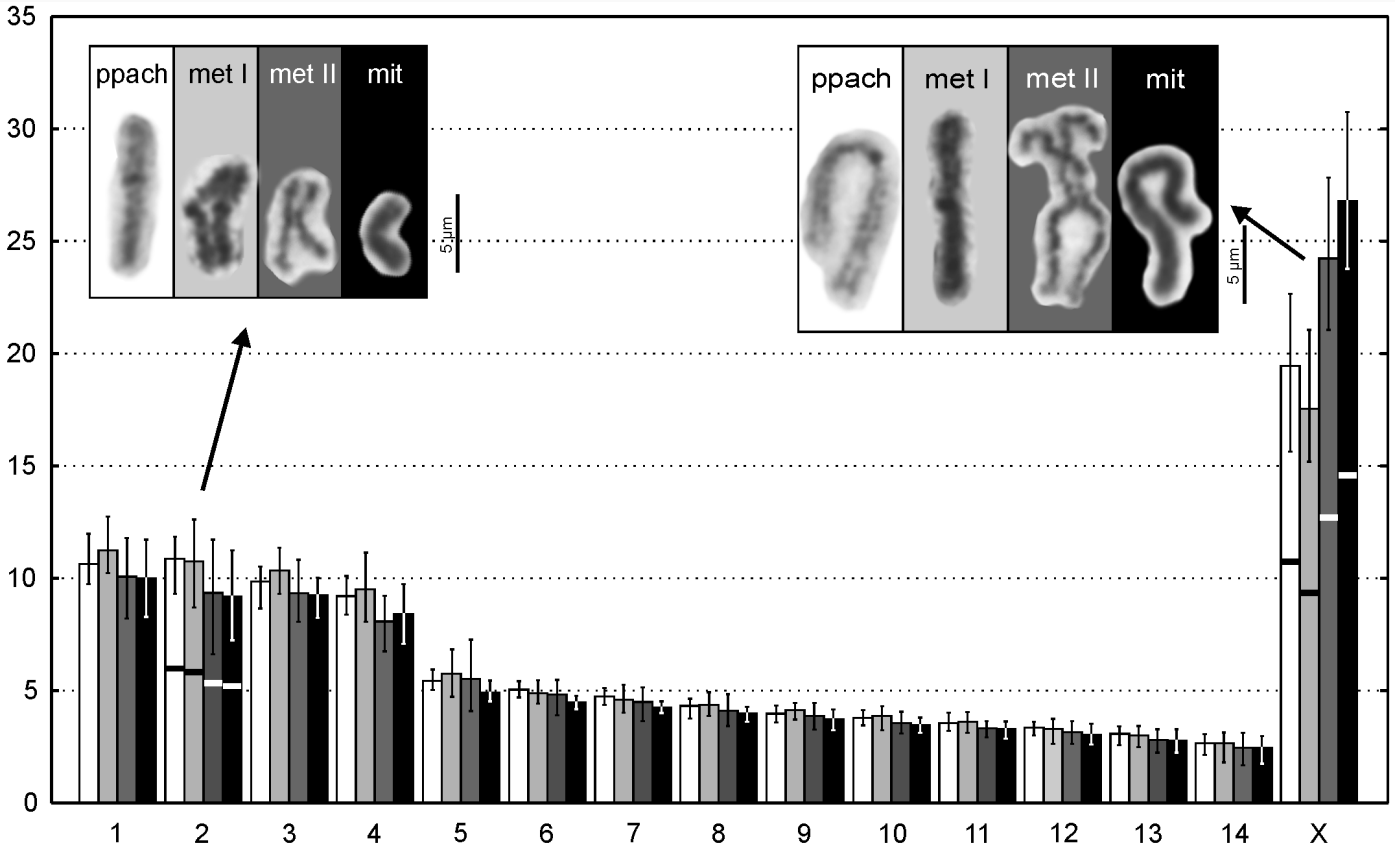
Ideograms of Chthonius (Chthonius) raridentatus cytotype I from Roče (y axis - % of the chromosome length of the haploid set). Comparison of different meiotic (ppach - postpachytene (white), met I - metaphase I (light grey), met II - metaphase II (dark grey)) and mitotic (mit - mitotic metaphase (black)) stages with examples of chromosomes 2 and X. Ideograms include min. - max. values and the centromeres are indicated only in metacentric chromosomes (all other chromosomes are acrocentrics).

The diploid number of chromosomes in all analysed individuals was 29 (Fig. 1e). Detailed analyses detected the existence of two cytotypes within this species. Cytotype I was found in most of the males, comprising 12 acrocentric autosome pairs, one metacentric pair (pair No. 2), and one subtelocentric pair (pair No. 5), and one metacentric X chromosome. Cytotype II was detected only in two individuals from different localities in northwest Slovenia. The karyotype is composed of 12 pairs of acrocentric and two pairs of metacentric autosomes (pairs No. 1 and 2), and one metacentric X (Fig. 1f). The karyotypes in both cytotypes showed a length differentiation of the autosomes; the first three pairs of autosomes are longer (roughly 3–3.5 ×) than the remaining chromosomes in the nucleus. In cytotype I, the first three autosomes are also considerably longer (RCLs 10.91%, 10.12% and 9.84%) than the remaining autosomes that gradually decrease in size, and X sex chromosome is the longest chromosome in the karyotype (Fig. 2). In cytotype II, RCLs of the three longest autosomes is 10.91%, 9.55% and 9.39% of the haploid set. The RCLs of the remaining autosomes gradually decrease from 7.39% to 2.90%, and the RCL of the X is 20.01%.

In cytotype I, we tested differences of the chromosome lengths and also arm ratio in biarmed chromosomes during distinct spiralization of several mitotic (mitotic metaphase (N = 44)) and meiotic stages (postpachytene (N = 14), metaphase I (N = 42), metaphase II (N = 29). We detected significant differences in two thirds of the comparisons among chromosomes (Suppl. material 1). The most considerable difference is noticed in the X chromosome during different stages (Fig. 2). It is probably an effect of different spiralization states of the X during meiosis and mitosis, also visible as different degrees of heteropycnosis (see paragraph below). The arm ratio of biarmed chromosomes (pair No. 2 and X) is significantly different only in few cases (Suppl. material 1) and the metacentric morphology has been detected during all analyzed stages.

**Figure 3.**
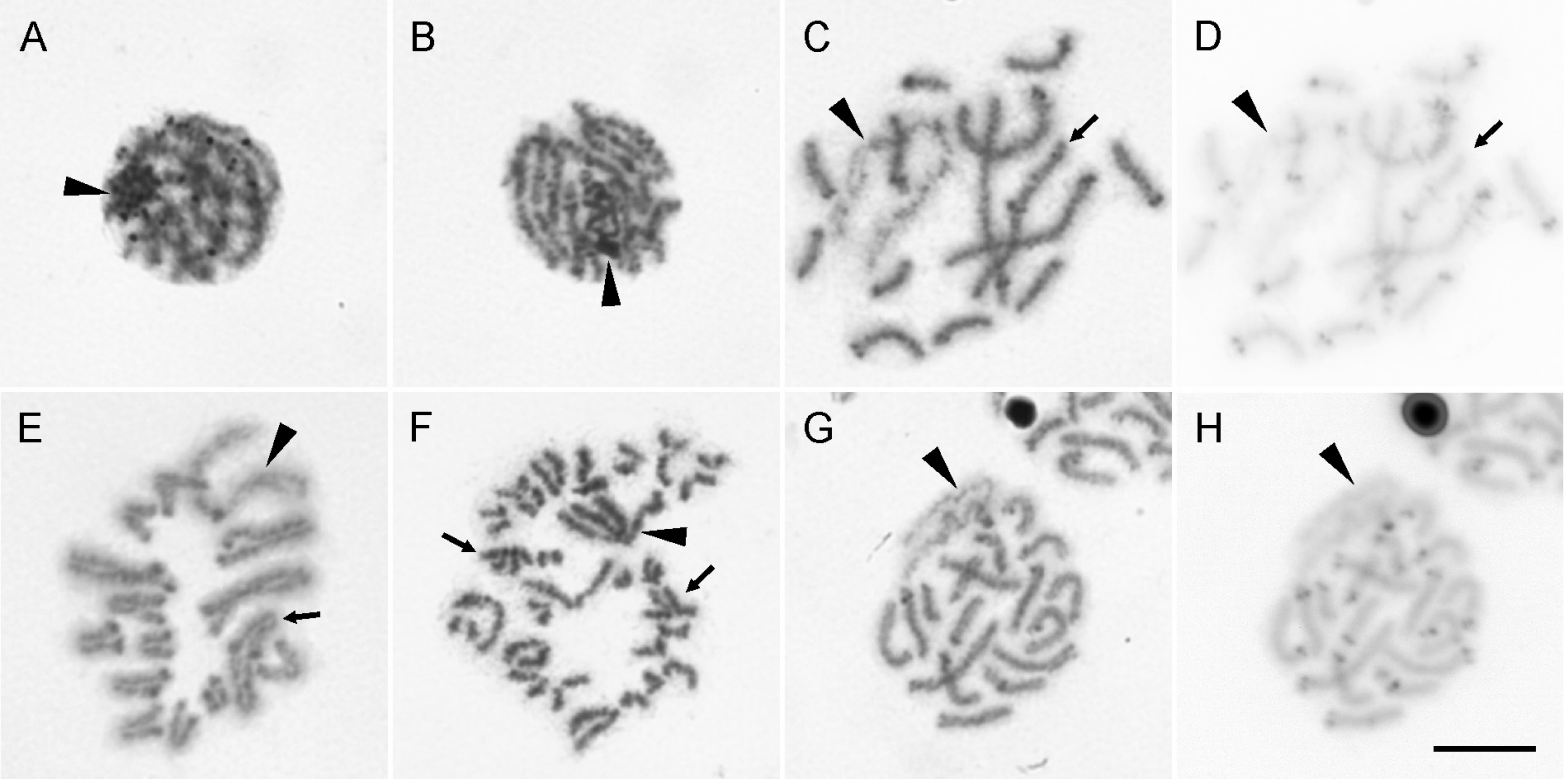
Meiotic chromosomes of Chthonius (Chthonius) raridentatus, cytotype I (**A–F**) and Chthonius (Ephippiochthonius) tetrachelatus (**G, H**). **A** zygotene **B** pachytene **C, D** postpachytene **E** metaphase I **F** metaphase II **G, H** postpachytene. Standard chromosomes stained with Giemsa (**A–C, E–G**) and the chromosomes after C-banding stained with DAPI (inverted) (**D, H**). Arrowheads indicate X sex chromosomes, arrows indicate metacentric autosomes. Scale bar = 10 µm.

During the meiosis X chromosome undergoes changes in condensation. During early prophase (leptotene-zygotene), the X forms a prominent spherical body and exhibits positive heteropycnosis (Fig. 3a). The body starts expanding during pachytene. The individual positively heteropycnotic arms of the X chromosome become visible, however they are still connected by their ends (Fig. 3b). During postpachytene, the sex chromosome becomes slightly negatively heteropycnotic (Fig. 3c). During metaphase I, all the chromosomes become isopycnotic except for prominent knobs in centromeric regions (Fig. 3e). These knobs are visible on the chromosomes from pachytene to metaphase I, but cannot be detected during metaphase II (Fig. 3f) and anaphase II. C-banding and analysis of cells at metaphase II of this species confirmed that constitutive heterochromatin is exclusively localized in these prominent knobs that correspond to the centromere regions (cf. Fig. 3c and 3d).

### Chthonius (Chthonius) rhodochelatus Hadži, 1933

Variability in chromosome morphology was detected in this species, resulting in two distinguishable cytotypes. The diploid number of chromosomes in cytotype I is 35. The karyotype comprises 15 acrocentric and two metacentric autosome pairs (pairs No. 2 and 13), and one metacentric X chromosome. No significant length differentiation of autosomes was detected. The RCLs of the autosomes gradually decrease from 6.25% to 3.24%. The RCL of the X is 22.43%. Cytotype II was detected in one male from a locality in the central Alps. This individual had a diploid set of 35 chromosomes (Fig. 1g). The karyotype comprises 15 acrocentric pairs, one metacentric pair (pair No. 13), and one submetacentric pair of autosomes (pair No. 14), with the X chromosome metacentric. The RCLs of the autosomes gradually decrease from 6.73% to 2.92%. The RCL of the X is 20.38%.

### Chthonius (Chthonius) tenuis L. Koch, 1873

**Figure 4.**
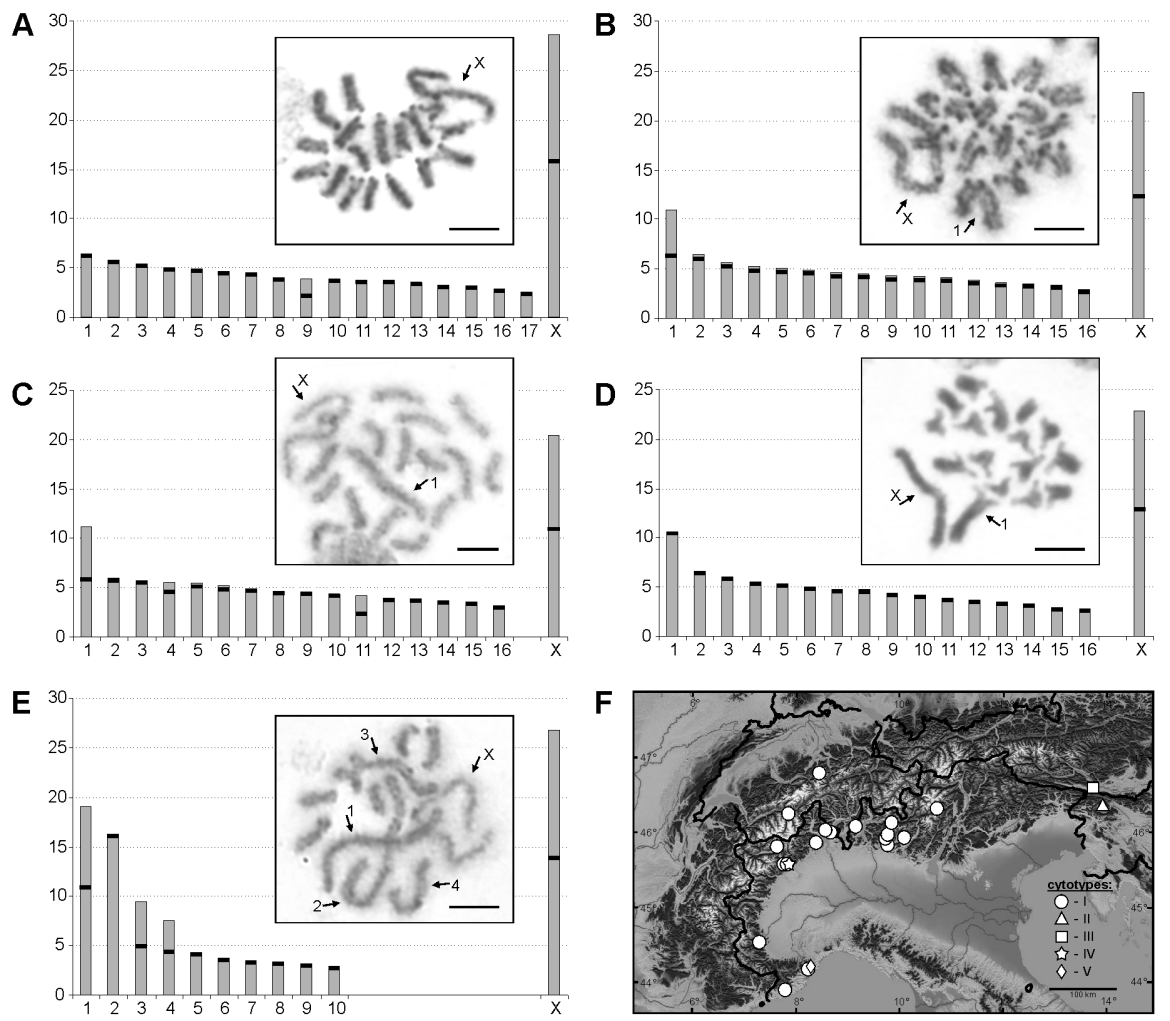
Ideograms of Chthonius (Chthonius) tenuis cytotypes (y axis - % of the chromosome length of the haploid set) and examples of chromosomes in postpachytene and metaphase I. **A** cytotype I (2n = 35, X0) **B** cytotype II (2n = 33, X0) **C** cytotype III (2n = 33, X0) **D** cytotype IV (2n = 33, X0) **E** cytotype V (2n = 21, X0) **F** distribution of cytotypes. Arrows indicate X sex chromosome and extra-large autosomes.

Variability in chromosome number and morphology was detected in this species, resulting in five distinguishable cytotypes (Fig. 4). Most individuals displayed cytotype I: 2n = 35 with 16 pairs of acrocentric and one pair of metacentric autosomes (pair No. 9), and one metacentric X chromosome (Fig. 4a). The RCLs of the autosomes gradually decrease from 6.41% to 2.65%. The RCL of X sex chromosome is 28.66%. Cytotypes II–IV have 2n = 33 and differ among themselves in the morphology of some autosome pairs. Cytotype II comprises 15 pairs of acrocentric and one pair of metacentric autosomes (pair No. 1) (Fig. 4b). The first autosomes pair (RCL 10.95%) is roughly two times longer than the rest. The RCLs of the remaining autosomes gradually decrease from 6.46% to 2.90%. Cytotype III comprises 13 pairs of acrocentric, two pairs of metacentric (pairs No. 1 and 11), and one pair of subtelocentric autosomes (pair No. 4) (Fig. 4c). The first pair of autosomes is significantly longer (RCL 11.18%) than the remaining autosomes whose RCLs gradually decrease from 5.95% to 3.13%. Cytotype IV comprises 16 acrocentric autosome pairs (Fig. 4d). The first autosome pair is significantly longer (RCL 10.61%) than the remaining autosomes whose RCLs gradually decrease from 6.59% to 2.72%. In all cytotypes with 2n = 33, the X is metacentric and the largest chromosome in the karyotype, with RCLs 22.87%, 20.44%, and 22.86% for cytotype II, III, and IV, respectively. Two male individuals of Chthonius (Chthonius) tenuis from Liguria showed a different chromosome number in the karyotype, namely 2n = 21. This cytotype V comprises seven pairs of acrocentric and three pairs of metacentric autosomes (pair No. 1, 3, and 4), and one metacentric X chromosome (Fig. 4e). In cytotype V, the two chromosomes of the first and the second pair are almost five times longer (RCLs 19.06% and 16.28%) than the other pairs. The two following autosome pairs are of medium size (RCLs 9.44% and 7.56%), whereas the remaining autosomes gradually decrease in RCL from 4.27% to 2.88% of the haploid set. The RCL of the X chromosome is 26.78%.

### Chthonius (Ephippiochthonius) boldorii Beier, 1934

**Figure 5.**
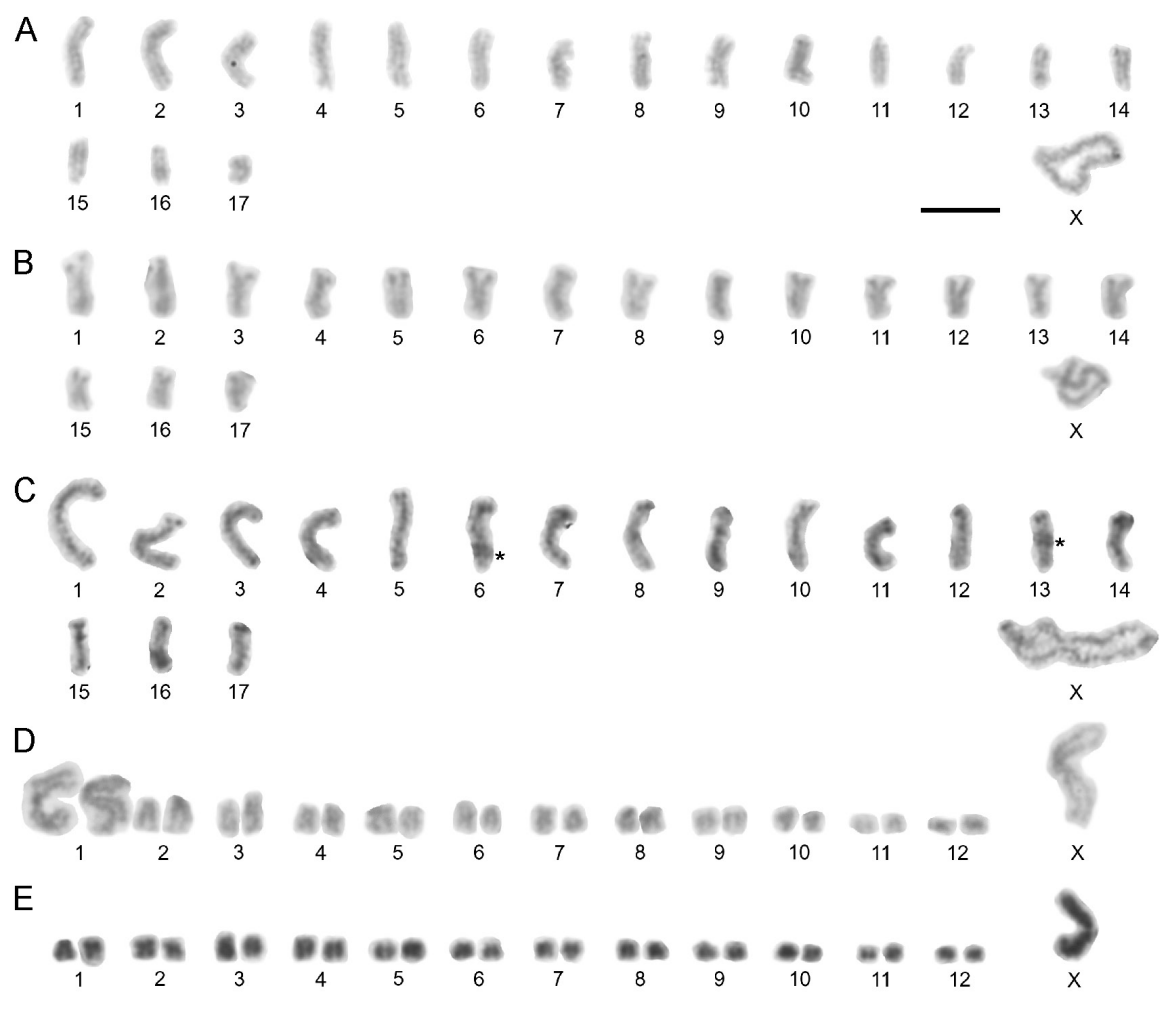
Karyotypes of Chthonius (Ephippiochthonius) and Chthonius (Globochthonius) males. **A** Chthonius (Ephippiochthonius) boldorii (2n = 35, X0), postpachytene **B** Chthonius (Ephippiochthonius) fuscimanus (2n = 35, X0), metaphase I **C** Chthonius (Ephippiochthonius) tetrachelatus (2n = 35, X0), postpachytene **D** Chthonius (Ephippiochthonius) nanus (2n = 25, X0), sister metaphases II **E** Chthonius (Globochthonius) poeninus (2n = 25, X0), mitotic metaphase. Asterisks indicate overlaps of chromosomes. Scale bar = 10 µm.

All examined individuals displayed 35 chromosomes in the diploid set (Fig. 5a). The karyotype of this species comprises 16 pairs of acrocentric and one pair of subtelocentric autosomes (pair No. 2), and one metacentric X sex chromosome. The autosomes gradually decrease in RCL from 7.38% to 2.43%. The RCL of the X is 19.99%.

**Figure 6.**
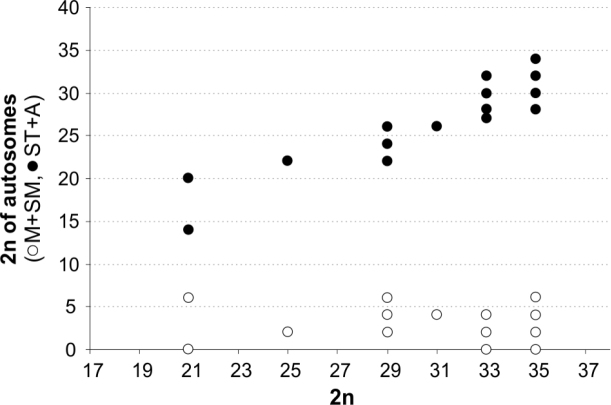
The proportion of biarmed (metacentric and submetacentric) and one-armed (subtelocentric and acrocentric) autosomes in karyotypes of the genus *Chthonius*. Data from Table 1.

### Chthonius (Ephippiochthonius) fuscimanus Simon, 1900

The diploid set of this species consists of 35 chromosomes (Fig. 5b). The karyotype comprises 17 acrocentric autosomes pairs and one metacentric X chromosome. The autosomes gradually decrease in RCL from 6.70% to 3.33%, and the RCL of the X is 18.86%.

### Chthonius (Ephippiochthonius) tetrachelatus Preyssler, 1790

The diploid set of this species comprises 35 chromosomes (Fig. 5c). They are 15 pairs of acrocentric, one pair of subtelocentric (pair No. 7), and one pair of submetacentric autosomes (pair No. 9); the X chromosome is metacentric. The autosomes gradually decrease in RCL from 7.45% to 2.96%, and the RCL of the X is 19.59%. C-banding confirmed the exclusive localization of constitutive heterochromatin in the centromere region (cf. Fig. 3g and 3h).

### Chthonius (Ephippiochthonius) nanus Beier, 1953

The diploid set consists of 25 chromosomes (Fig. 5d). The karyotype possesses 11 acrocentric autosome pairs and one metacentric pair (pair No. 1), and one metacentric X chromosome. The metacentric pair of autosomes is significantly longer (RCL 20.91%) than the remaining autosomes whose RCLs decrease from 6.26% to 2.96%. The RCL of the X is 31.03%.

### Chthonius (Globochthonius) poeninus Mahnert, 1979

The diploid set of this species consists of 25 chromosomes (Fig. 5e). The karyotype comprises of 11 pairs of acrocentric and one pair of metacentric autosomes (pair No. 2), and one metacentric X sex chromosome. The autosomes gradually decrease in RCL from 8.04% to 3.37%, and the RCL of the X is 33.74%.

## Discussion

### Chromosomal characteristics of the genus *Chthonius*

Only 11 species of Chthoniidae have been studied so far, eight of them belonging to the genus *Chthonius* from Romania, Czech Republic, and Greece (Šťáhlavský and Král 2004) (Table 1). Analyses of 11 species from the Alps fully confirm previously detected cytogenetic characteristics of the genus. Pseudoscorpions from the Alps present monocentric chromosomes, similar to other representatives of the genus as well as all other pseudoscorpion taxa that have been cytogenetically analysed (e.g. Šťáhlavský and Král 2004, Šťáhlavský et al. 2006). Achiasmatic meiosis has been confirmed in males of the genus *Chthonius*, which is probably characteristic for the entire family Chthoniidae (Šťáhlavský and Král 2004). This meiosis type is otherwise known within the class Arachnida only in scorpions (e.g. Schneider et al. 2009), spiders from the families Dysderidae and Segestriidae (Benavente and Wettstein 1980) and mites from the superfamily Hydrachnellae (Oliver 1977). Given that groups presenting achiasmatic meiosis are not closely related (e.g. Sharma et al. 2014), multiple independent origins of achiasmatic meiosis within arachnids could be assumed. Another characteristic specific to the genus *Chthonius* is the sex chromosome system. The typical sex chromosome system in the family Chthoniidae is X0 (Šťáhlavský and Král 2004), which was detected in all species analysed in the present study. The X sex chromosome is always metacentric and is the longest chromosome in the karyotype for all species in the genus *Chthonius*. The sex chromosome system with a large metacentric chromosome X has been detected in most pseudoscorpion families that have been cytogenetically analysed (Chernetidae, Geogarypidae, Garypinidae, Olpiidae, Atemnidae) (Šťáhlavský et al. 2005, 2006, 2012). Metacentric morphology and large size of X sex chromosomes have been documented within arachnids also in different species from different groups of spiders (e.g. Král et al. 2006, 2013). Furthermore, extremely large metacentric X sex chromosomes is also known in some beetles from the family Chrysomelidae (Insecta, Coleoptera) (e.g. Almeida et al. 2009). It is evident that this type of X chromosome would have originated independently by different evolutionary mechanisms. However, there are only few exceptions to the morphology of the X chromosome in pseudoscorpions, namely one population of the species *Olpium pallipes* (Lucas, 1849) (Olpiidae) (Šťáhlavský et al. 2006) and two neotropic species *Semeiochernes armiger* (Balzan, 1892) and *Cordylochernes scorpioides* (Linnaeus, 1758) (Chernetidae) (Šťáhlavský et al. 2009). This evidence supports the assumption that the X0 sex chromosome system with large metacentric X is the plesiomorphic state in pseudoscorpions (e.g. Troiano 1990, 1997, Šťáhlavský et al. 2012).

The C-banding analyses performed in this study represent the first time that this procedure is applied in pseudoscorpions. Constitutive heterochromatin was only detected in the centromere regions. Blocks of heterochromatin located on different parts on the chromosome, known from some araneomorph spiders (Král et al. 2006), have not been detected. The concentration of constitutive heterochromatin in the centromere region could represent an ancestral state in this group, similar to that hypothesized in spiders (Rodríguez-Gil et al. 2007). The C-banding also confirmed the hypothesis that the prominent heteropycnotic blocks on chromosomes, visible in the early stages of meiosis, correspond to centromeres (Šťáhlavský et al. 2006). The results also indicate that the X is formed mainly of euchromatin outside of the centromere region, and the positive heteropycnosis during the early phases of meiosis is caused by intensive condensation, similarly as reported in wolf spiders (Dolejš et al. 2010).

### Karyotype evolution of the genus *Chthonius*

Overall, the pseudoscorpions are represented by a great variety of chromosome numbers from 7 in Olpiidae to 143 in Atemnidae (Šťáhlavský et al. 2012). However, there is a much narrower range within individual families. For example, the typical range of the diploid number for a specific family is: 2n = 7–23 in Olpiidae, 2n = 15–23 in Geogarypidae, 2n = 16–67 in Neobisiidae, 2n = 47–73 in Chernetidae, and 2n = 65–143 in Atemnidae (see Šťáhlavský 2016). The results of this study confirm that the variability in chromosome number in the genus *Chthonius* is in agreement with previous findings (Šťáhlavský and Král 2004). The diploid number in males ranged from 21–35 (most frequently 35) and the acrocentric chromosomes are the most common morphology in the karyotype (see Table 1). Šťáhlavský and Král (2004) suggested that this chromosome number, acrocentric morphology of the chromosomes, and their gradual decrease in size are the ancestral conditions for species of this genus. The findings of this study confirm the assumption in Chthonius (Chthonius) carinthiacus and Chthonius (Ephippiochthonius) fuscimanus with 2n = 35 species presenting only acrocentric chromosomes. In other species with a diploid number 2n = 35 and a majority of acrocentric chromosomes, the presence of different morphological types of autosomes has also been detected, probably as a result of pericentric inversions. Šťáhlavský and Král (2004) also hypothesized that centric and tandem fusions play an important role in the karyotype evolution of the genus *Chthonius*, leading to a decrease in the number of chromosomes and a change of their morphology from uniarmed to biarmed. The exact mechanism and direction of the karyotype evolution of pseudoscorpions remain unknown. However, the abundant frequency of karyotypes with 35 chromosomes (Table 1) could indicate that this number corresponds to the ancestral state. Similar changes of the number, morphology, and size of the chromosomes linked to both centric and tandem fusions can be observed in the different cytotypes of Chthonius (Chthonius) tenuis (Fig. 4a–e). We detected several cytotypes (II, III and IV) with the same diploid number of 33 in this species. The number presumably decreased from the hypothetic ancestral state of 2n = 35 (cytotype I, Fig. 4a) by means of centric (cytotype II, III, Fig. 4b, c) or tandem (cytotype IV, Fig. 4d) fusions. Independent (centric or tandem) fusions or subsequent pericentric inversions may produce a different morphology of extra large autosomes. Accumulation of chromosome fusions occurred in cytotype V decreasing the number to 2n = 21 and leading to differentiation of autosomes into three categories: large, medium, and small (Fig. 4e). In Chthonius (Chthonius) alpicola species, with a chromosome number lowered to 2n = 21 and presence of only acrocentric chromosomes in the karyotype (Fig. 1a), multiple tandem fusions presumably played a key role in the process of lowering the chromosome number. From the comparison of the frequency of morphologic types of chromosomes in the karyotypes of the genus *Chthonius* (Fig. 6), it is apparent that the frequency of metacentric and submetacentric chromosomes does not vary considerably, but the number of acrocentric and subtelocentric autosomes decreases in karyotypes with lower chromosome numbers. This phenomenon could be explained because of tandem fusions, or potentially as a result of a centric fusion subsequently accompanied by a pericentric inversion. However, for precise understanding of these mechanisms, the use of additional cytogenetic tools would be needed in for the exact identification of homologous segments of the chromosomes and detection of particular chromosome rearrangements leading to changes in both chromosome morphology and size (e.g. Nie et al. 2012). Unfortunately, the exact direction of karyotype evolution within the genus *Chthonius* is also not possible to determine without the knowledge of the phylogenetic relationships among the *Chthonius* species, which are currently unknown.

### Cryptic species or intraspecific variability of karyotypes?

Karyotypes of pseudoscorpions show considerable differences among species within all analysed families (see Šťáhlavský 2016), and therefore cytogenetic data have great potential for taxonomic application in the order. The usefulness of karyology has been demonstrated in the genus *Roncus* L. Koch, 1873. This mainly European group, with more than 140 described species (Harvey 2013), usually exhibits similar external morphology (e.g. Gardini 1991). However, the karyotypes of very morphologically similar species differ in diploid number, chromosome morphology, and sex chromosome systems (X0 and XY) (e.g. Troiano 1990). Substantial differences in karyotypes were used to distinguish *Roncus montsenyensis* Zaragoza and Šťáhlavský, 2008 (2n = 16) from *Roncus cadinensis* Zaragoza, 2007 (2n = 38), which are morphologically very similar (Zaragoza and Šťáhlavský 2008). Unfortunately, the use of cytogenetics in cryptic species detection in pseudoscorpions is complicated due to the limited amount of data available for comparison among different species. Furthermore, different degrees of intraspecific karyotype variability can represent a problem to determine and distinguish the existence of cryptic or isomorphic species. These challenges, observed in the genus *Chthonius* from the Alps, are also known from various arachnid groups (e.g. Tsurusaki 1985, Řezáč et al. 2007, Schneider et al. 2009), and many other organisms (e.g. Duffy et al. 2008, Severns and Liston 2008, Dincǎ et al. 2011, Sadílek et al. 2013, Sember et al. 2015).

Our data completely agree with described karyotypes of Chthonius (Ephippiochthonius) fuscimanus and Chthonius (Chthonius) carinthiacus (misidentified see Christophoryová et al. 2012) from Central Europe (both species 2n = 35, only acrocentric autosomes) (Šťáhlavský and Král 2004). Small differences between karyotypes of Chthonius (Ephippiochthonius) tetrachelatus from Central Europe (Šťáhlavský and Král 2004) and from the Alps (present study) may be an artefact of the quality of the preparations. Furthermore, the precision of visualizing the centromere position, using C-banding in this study, was likely better. Karyotype similarity from distant localities within these three species suggests that the use of these approaches may also contribute to characterize pseudoscorpion species very well.

In contrast to these findings, we identified different cytotypes in more abundant material of four species of the subgenus *Chthonius* from the Alps (Table 1). Only in Chthonius (Chthonius) ischnocheles and Chthonius (Chthonius) rhodochelatus, different cytotypes were complemented by visible morphological differences in surface granularity and pedipalp size. These particular morphological differences would potentially allow us to treat the cytotypes as new cryptic species. Nevertheless, standard morphological characteristics overlap substantially among the cytotypes in both Chthonius (Chthonius) raridentatus and Chthonius (Chthonius) tenuis. The interpretation of the variability in this case is not trivial. The Chthonius (Chthonius) tenuis cytotypes may present both intraspecific variability and a mix of different levels of speciation. Specific cytotypes from different parts of the Alps have been reported in species with large distribution (e.g. Capanna and Riscassi 1978, Zima et al. 1996), but the difference between the highly derived cytotype V (2n = 21) and the remaining cytotypes most likely represents a strong reproductive barrier between them. Thus, cytotype V may represent a cryptic species without evident morphological differentiation. The fact that cytotype I spatially overlaps cytotype V (Fig. 4f) coupled with the fact that no anomalies during meiosis have been detected in the studied material, suggests an absence of hybridization between them and further supports the cryptic species status of cytotype V (e.g. King 1993). It should be noted that material used in the present study is probably not substantial enough to rule out the existence of hybrids and more investigation is needed before any taxonomic changes can be made. The putative chromosomal speciation of *Chthonius* in the Alps may be owed to the dispersal limitation of the genus. Chromosomal rearrangements may be fixed in certain areas with limited gene flow and consequently cause hybrid sterility among different cytotypes. Alternatively, the environments of the Alps may influence chromosome rearrangement that favours higher fitness for specific environmental conditions (see e.g. Faria and Navarro 2010). Additional sampling, comprehension of phylogenetic relationships among the analysed species, and knowledge of the genetic structure of their populations are needed for the full understanding of karyotype variability and its role in the speciation of the genus *Chthonius* in the Alps.

## Supplementary materials

Supplementary material 1 Table S1 Data type: karyometric data Explanation note: Comparison of karyometric data of particular chromosomes using t-test: t values (above the diagonal), p-values (below the diagonal), significant differences (α = 0.05) in bold. This dataset is made available under the Open Database License (http://opendatacommons.org/licenses/odbl/1.0/). The Open Database License (ODbL) is a license agreement intended to allow users to freely share, modify, and use this Dataset while maintaining this same freedom for others, provided that the original source and author(s) are credited.Jana Kotrbová, Vera Opatova, Giulio Gardini, František Šťáhlavský
